# Dapagliflozin & pioglitazone combination therapy in T2DM with or without MASLD - a systematic review and meta-analysis: PRO-2 study

**DOI:** 10.3389/fcdhc.2026.1733995

**Published:** 2026-07-20

**Authors:** Shashank Joshi, Shambo Samrat Samajdar, Arvind Gupta, Mithun Bhartia, Sailesh Lodha, Supratik Bhattacharyya, Krishna Seshadri, Lakshmi Nagendra, A. G. Unnikrishnan, Dakshata Padhye, Lakshmi Nalini Kopalle, Ashok Kumar Das, Om Lakhani, Jothydev Kesavadev, Ameya Joshi, Sambit Das, Smriti Gadia, Thamburaj Anthuvan

**Affiliations:** 1 Department of Endocrinology, Lilavati Hospital and Research Centre, Mumbai, Maharashtra, India; 2 Department of Pharmacology, JMN Medical College and Hospital, Ranaghat, West Bengal, India; 3 RHL–Rajasthan Hospital, Jaipur, Rajasthan, India; 4 Apollo Hospital, Guwahati, Assam, India; 5 Department of Endocrinology, Eternal Hospital, Jaipur, Rajasthan, India; 6 SKN Diabetes and Endocrine Centre, Kolkata, West Bengal, India; 7 Chennai Diabetes and Endocrine Clinic, Apollo Hospitals, Chennai, Tamil Nadu, India; 8 Department of Endocrinology, JSS Medical College, JSS Academy of Higher Education and Research, Mysore, Karnataka, India; 9 Chellaram Diabetes Institute, Pune, Maharashtra, India; 10 Conquer Diabetes Clinic, Mumbai, Maharashtra, India; 11 Fernandez Foundation Unit 1, Hyderabad, Telangana, India; 12 Mahatma Gandhi Medical College and Research Centre, Pondicherry, India; 13 Zydus Hospital, Ahmedabad, India; 14 Jothydev’s Diabetes Research Centre, Trivandrum, Kerala, India; 15 Bhaktivedanta Hospital & Research Institute, Mumbai, Maharashtra, India; 16 Department of Endocrinology, Kalinga Institute of Medical Sciences, Bhubaneswar, Odisha, India; 17 Scientific Services, USV Pvt Ltd, Mumbai, Maharashtra, India; 18 Sales & Marketing and PhD Scholar, USV Private Limited and Pimpri Chinchwad Education Trust (PCET)’s S.B. Patil Institute of Management (Savitribai Phule Pune University), Mumbai, Maharashtra, India

**Keywords:** combination therapy, dapagliflozin, MASLD, NAFLD, pioglitazone, SRMA, T2DM

## Abstract

**Aims:**

To evaluate the efficacy and safety of dapagliflozin plus pioglitazone versus comparators on metabolic control and hepatic steatosis in adults with type 2 diabetes mellitus (T2DM), with or without metabolic dysfunction-associated steatotic liver disease (MASLD).

**Methods:**

We conducted a systematic review and meta-analysis of randomized and observational studies in PubMed, Embase, Cochrane Central, Scopus, Web of Science with a search updated through January 2026, following peer review. Eligible studies enrolled adults with T2DM with or without MASLD, receiving dapagliflozin plus pioglitazone in comparison with standard care or monotherapy, or as pooled-arm exploratory estimates. The primary outcome was glycemic control (HbA1c and fasting and postprandial glucose levels). Secondary outcomes included body weight, insulin resistance indices, related liver outcomes (liver fat content, NAFLD activity score [NAS], and steatohepatitis resolution), liver enzymes, non-invasive fibrosis indices, lipid parameters, and safety outcomes.

**Results:**

Thirteen studies (n = 1411) met the inclusion criteria. Dapagliflozin–pioglitazone significantly improved glycemic control, with a reduction in HbA1c (mean difference −0.49%; 95% CI −0.64 to −0.34), and Fasting Blood Glucose (−11.96 mg/dL; 95% CI −16.30 to −7.62) and Post-Prandial Glucose (−21.54 mg/dL; 95% CI −30.84 to −12.24). Hepatic outcomes also improved, with reductions in liver fat content (standardized mean difference −0.42; 95% CI −0.61 to −0.22) and NAS (standardized mean difference −0.39; 95% CI −0.56 to −0.22), and higher rates of steatohepatitis resolution (risk ratio 1.48; 95% CI 1.13–1.92). Certainty of evidence was moderate for glycemic and steatosis outcomes and low for histological endpoints.

**Conclusions:**

Dapagliflozin–pioglitazone combination therapy was associated with improvements in glycemic control and non-invasive markers of hepatic steatosis in adults with T2DM, with or without MASLD, while maintaining a favorable safety profile. Certainty of evidence was moderate for metabolic and steatosis outcomes and low for histological endpoints. Given the predominance of surrogate hepatic endpoints and limited biopsy-confirmed data, these findings should be interpreted with appropriate caution.

**Systematic review registration:**

https://www.crd.york.ac.uk/prospero/display_record.php?RecordID=1117019, identifier CRD420251117019.

## 1 Introduction

Type 2 diabetes mellitus (T2DM) has emerged as a major global public health challenge, affecting an estimated 589 million adults worldwide. In 2024, diabetes was directly responsible for approximately 3.4 million deaths, and projections suggest that the global burden will increase to nearly 853 million individuals by 2050. India contributes substantially to this burden, with nearly 89.8 million people living with diabetes by 2024 (1). This condition is associated with considerable morbidity and mortality, driven by a wide spectrum of complications involving multiple organ systems. These include well-recognized microvascular complications such as retinopathy, neuropathy, and nephropathy, as well as macrovascular complications including cerebrovascular disease, peripheral arterial disease, and cardiovascular disease (2, 3). Although advances in diabetes care have improved outcomes for several traditional complications, increasing attention is now being directed toward emerging, non-traditional complications that further amplify cardiometabolic risk.

Metabolic dysfunction–associated steatotic liver disease (MASLD) is increasingly being recognized as a common and clinically important complication of diabetes. MASLD shows a strong bidirectional association with obesity and T2DM, with insulin resistance and metabolic dysregulation serving as the shared pathogenic drivers (4–6). As the global prevalence of T2DM and obesity continues to rise, the prevalence of MASLD has increased in parallel, now affecting a substantial proportion of adults worldwide. Beyond its hepatic manifestations, MASLD contributes to a significant clinical and socioeconomic burden, including impaired quality of life, fatigue, reduced work productivity, and increased healthcare utilization (7). These features position MASLD not merely as a coincidental comorbidity, but as an integral component of the metabolic disease spectrum associated with diabetes.

MASLD is a major, yet often under-recognized, driver of cardiovascular disease, which remains the leading cause of mortality in this population. Large meta-analyses and population-based cohort studies have demonstrated approximately 60–100% higher rates of fatal and nonfatal cardiovascular events among individuals with MASLD than among metabolically healthy controls, with cardiovascular risk escalating progressively across advancing fibrosis stages (8–10). These observations firmly establish MASLD as a systemic cardiometabolic disorder, rather than an isolated hepatic condition. Consequently, MASLD warrants systematic identification and integrated risk management that extends beyond liver-directed endpoints to include proactive control of dyslipidemia, hypertension, and glycemic burden, alongside structured lifestyle intervention.

While structured lifestyle modifications, including dietary intervention and physical activity, remain the cornerstone of MASLD management, long-term adherence and sustained metabolic benefits are difficult to achieve in routine clinical practice (7). In individuals with established T2DM, lifestyle measures alone are frequently insufficient to counteract persistent insulin resistance, glucotoxicity, and ectopic lipid accumulation, which drive both hepatic and cardiometabolic disease progression. This therapeutic gap has heightened interest in pharmacological strategies that can simultaneously address hyperglycemia, excess adiposity, and downstream hepatic injury, particularly in high-risk metabolic populations where multidomain metabolic control is needed.

Sodium–glucose co-transporter 2 inhibitors (SGLT2i) are now well established in the management of T2DM, owing to their durable glycemic efficacy and proven cardiovascular and renal benefits. Beyond lowering glucose levels, these agents promote modest weight loss, improve insulin sensitivity, and reduce cardiometabolic risk, making them particularly attractive in patients with complex metabolic diseases. Increasing evidence indicates that SGLT2i also exert favorable hepatic effects, including reductions in liver enzymes, hepatic steatosis, and fibrosis-related indices, with real-world studies suggesting a lower risk of cirrhosis compared to dipeptidyl peptidase-4 inhibitors. Indian studies and regional consensus statements further support their use in patients with T2DM and coexisting fatty liver disease, highlighting their relevance as early therapeutic options in this population (11).

Dapagliflozin is a commonly used SGLT2i in routine clinical practice for the treatment of T2DM. Beyond its established glucose-lowering efficacy, a recent meta-analysis demonstrated that dapagliflozin treatment was associated with significant reductions in alanine aminotransferase (ALT) and aspartate aminotransferase levels (AST), along with improvements in glycemic control. Additional benefits include reduction in body weight, blood pressure, and insulin resistance, all of which are relevant to the metabolic milieu that underpins MASLD (12). These data support dapagliflozin as a diabetes therapy with consistent downstream metabolic and hepatic signals, providing a rational foundation for its evaluation in combination strategies.

Pioglitazone, a thiazolidinedione (TZD), improves insulin sensitivity through the activation of peroxisome proliferator-activated receptor-γ (PPAR-γ), with downstream effects on adipose tissue function, free fatty acid flux, and hepatic insulin signaling. Clinical trials have consistently shown that pioglitazone reduces hepatic fat content, improves transaminase levels, and promotes the resolution of steatohepatitis, with evidence of fibrosis improvement in selected populations, including individuals with prediabetes or without diabetes. These hepatic benefits have led to the conditional endorsement of pioglitazone in international and regional guidelines for the management of MASLD in patients with T2DM (11, 13–15). However, concerns related to weight gain, fluid retention, and long-term tolerability have limited broader uptake, highlighting the need for strategies that preserve hepatic efficacy while mitigating adverse metabolic effects.

Combining an SGLT2i with pioglitazone offers a mechanistically complementary approach that targets multiple drivers of metabolic dysfunction in T2DM and MASLD. While SGLT2 inhibition promotes glucosuria, weight reduction, and improved cardiometabolic profiles, pioglitazone enhances insulin sensitivity and exerts direct hepatic benefits, including reduction in steatosis and inflammatory activity. Importantly, SGLT2i may mitigate pioglitazone-associated weight gain and fluid retention through osmotic diuresis and caloric loss, thereby improving the overall tolerability. Emerging clinical and real-world evidence suggests that this combination can deliver additive or synergistic improvements in glycemic control, hepatic steatosis, and cardiometabolic risk while preserving an acceptable safety profile (11, 16, 17). This complementary balance provides a strong biological and clinical rationale for systematic evaluation of dapagliflozin–pioglitazone combination therapy in patients with T2DM and coexisting or probable MASLD.

Recent advances have expanded the therapeutic landscape of MASLD to include advanced fibrosis. The phase 3 MAESTRO-NASH trial demonstrated histological improvement with Resmetirom in patients with fibrosis stages F2–F3, whereas the ESSENCE trial confirmed biopsy-proven benefits of once-weekly semaglutide in metabolic dysfunction-associated steatohepatitis (MASH) with similar fibrosis stages (18, 19). A phase 2b trial of the dual GLP-1/GIP receptor agonist Tirzepatide demonstrated histologic improvements in steatohepatitis and fibrosis (20). However, these agents are primarily indicated for advanced disease, require injectable administration, and may be limited by cost and accessibility in many healthcare settings, underscoring the continued clinical relevance of effective, scalable oral combination strategies, such as dapagliflozin plus pioglitazone.

Despite growing evidence supporting the individual metabolic and hepatic benefits of dapagliflozin and pioglitazone, existing systematic reviews have largely evaluated these agents as monotherapies or against placebo, and have not comprehensively assessed their combined effects. Although individual clinical trials and real-world studies have explored the glycemic or hepatic outcomes of SGLT2i and TZDs, to our knowledge no systematic review or meta-analysis has specifically examined the additive impact of dapagliflozin–pioglitazone combination therapy in adults with T2DM and coexisting or probable MASLD, including data from the Indian context. To address this gap, the present study was conducted as PRO-2 within the Pioglitazone–SGLT2i Combination Research Outcomes (PRO) series, following the randomized Phase 3 PRO-1 clinical trial, as a structured evidence synthesis initiative to evaluate combination therapies in metabolically complex populations (21). This systematic review and meta-analysis synthesized evidence from randomized controlled trials and real-world observational studies published between 2012 and 2026, comparing the glycemic, hepatic, and metabolic efficacy and safety of dapagliflozin–pioglitazone combination therapy with other antidiabetic regimens in adults with T2DM and MASLD.

## 2 Methods

### 2.1 Study design and protocol registration

This systematic review and meta-analysis was conducted in compliance with the Preferred Reporting Items for Systematic Reviews and Meta-Analyses (PRISMA) 2020 guidelines and was registered prospectively in the International Prospective Register of Systematic Reviews (PROSPERO; ID: CRD420251117019). Following peer review, the literature search was updated to include newly published eligible studies, without modification of pre-specified objectives or outcomes. The primary objective of this study was to assess the efficacy of dapagliflozin combined with pioglitazone in adults with T2DM with or without MASLD. The primary outcome of interest was glycemic control, assessed by glycated hemoglobin (HbA1c), fasting blood glucose (FBG), and postprandial glucose (PPG). All other metabolic, hepatic, anthropometric, and safety endpoints were evaluated as the secondary outcomes.

### 2.2 Information sources and search strategy

A multidisciplinary and free-text search was conducted across PubMed, Embase, Cochrane Central, Scopus, Web of Science to ensure maximal recall of studies related to MASLD terminology and legacy non-alcoholic fatty liver disease (NAFLD) and non-alcoholic steatohepatitis (NASH) terms combined specifically with dapagliflozin and pioglitazone, including relevant synonyms and TZD-related concepts. The searches were updated following a peer review to capture newly published eligible studies. The strategy employed Boolean operators (AND/OR), truncation, and wildcards adapted to individual database syntax, applied English language and human study limits only, and included manual screening of reference lists and linked trial registry records, including associated conference reports, to minimize selection bias while reflecting real-world reliance on non-invasive hepatic assessment tools such as ultrasound and transient elastography with Controlled Attenuation Parameter (CAP).

### 2.3 Eligibility criteria

Studies were considered eligible if they were original research articles published between July 2012 and January 2026 that included adult patients diagnosed with T2DM receiving pharmacological therapy. Eligible studies evaluated pioglitazone combined with dapagliflozin, either in comparative designs against standard of care, placebo, or active comparators or as pooled-arm exploratory estimates reporting pre- and post-treatment effects. Studies are required to report the efficacy outcomes related to glycemic control, including HbA1c, FBG, and PPG. Additional outcomes of interest included lipid parameters, liver function tests, liver disease indices, liver enzymes, body weight, and safety outcomes, such as adverse drug reactions (ADRs), hypoglycemia, and cardiovascular events.

Studies focusing on type 1 diabetes, animal or *in vitro* experiments, reviews, editorials, letters, case reports, and pediatric populations as well as studies with insufficient or non-extractable outcome data and non-English publications were excluded. After duplicate removal, two independent reviewers screened the titles and abstracts, followed by a full-text assessment of potentially eligible articles. Disagreements were resolved through discussions or third-party adjudication. Data were extracted using a standardized form capturing the study design, patient characteristics, treatment regimens, hepatic outcomes, glycemic measures, and reported adverse events.

### 2.4 Risk of bias assessment

The risk of bias was assessed independently by two reviewers using standardized tools. Randomized controlled trials were evaluated using the Cochrane Risk of Bias 2 (RoB 2) tool, and observational studies were evaluated using the Risk of Bias in Non-randomized Studies of Interventions (ROBINS-I) tool. Most randomized trials were judged to have a low risk of bias; observational studies demonstrated a moderate risk, primarily due to confounding factors. See **Appendix Figure A1** for the risk of bias summary and **Appendix Table A1** for Grading of Recommendations Assessment, Development and Evaluation (GRADE) certainty ratings.

### 2.5 Certainty of the evidence

The certainty of evidence for each predefined outcome was evaluated using the GRADE approach, considering study limitations (risk of bias), inconsistency, indirectness, imprecision, and potential publication bias. Two reviewers independently rated the certainty of all key outcomes, with discrepancies resolved by consensus and third-author adjudication. Overall, the certainty of evidence was moderate for glycemic and body weight outcomes and low to moderate for lipid parameters, primarily due to heterogeneity across studies and limited sample sizes in certain subgroups. Detailed GRADE judgments and evidence profiles are provided in **Table A1** of the **Appendix**.

The inclusion of both randomized and observational studies was pre-specified to maximize evidence capture given the limited RCT landscape. Observational studies are subject to confounding by indication, selection bias, and variable outcome ascertainment, which may influence pooled estimates. To address this, design-appropriate risk of bias tools (RoB 2 for RCTs; ROBINS-I for observational studies) and GRADE-based certainty ratings were applied. Pooled estimates should be interpreted in light of the contributing study designs.

### 2.6 Synthesis methods

A qualitative narrative synthesis was conducted, along with quantitative pooling (meta-analysis). Heterogeneity was assessed using the I² statistic. The following interpretation of I² values was used: 0–25%, low heterogeneity; 25–50%, moderate heterogeneity; 50–75%, substantial heterogeneity; >75%, considerable heterogeneity. We used RevMan version 5.4 for conducting the meta-analysis. Statistical significance was set at p-value < 0.05. Publication bias was assessed visually using funnel plots. A formal Egger’s regression test was not performed because fewer than 10 studies were included in most outcomes. All quantitative syntheses were performed using the DerSimonian–Laird random-effects model to account for inter-study heterogeneity.

One randomized phase 3 trial reported pooled-arm exploratory glycemic outcomes for dapagliflozin plus pioglitazone without a parallel untreated comparator. In line with prior real-world and longitudinal cohort studies, this study was included as a single-arm pre–post estimate for glycemic outcomes only (HbA1c, FBG, and PPG). No assumptions were made regarding comparator changes, and the study did not contribute to hepatic, lipid, or safety outcome syntheses.

## 3 Results

**Figure 1:**
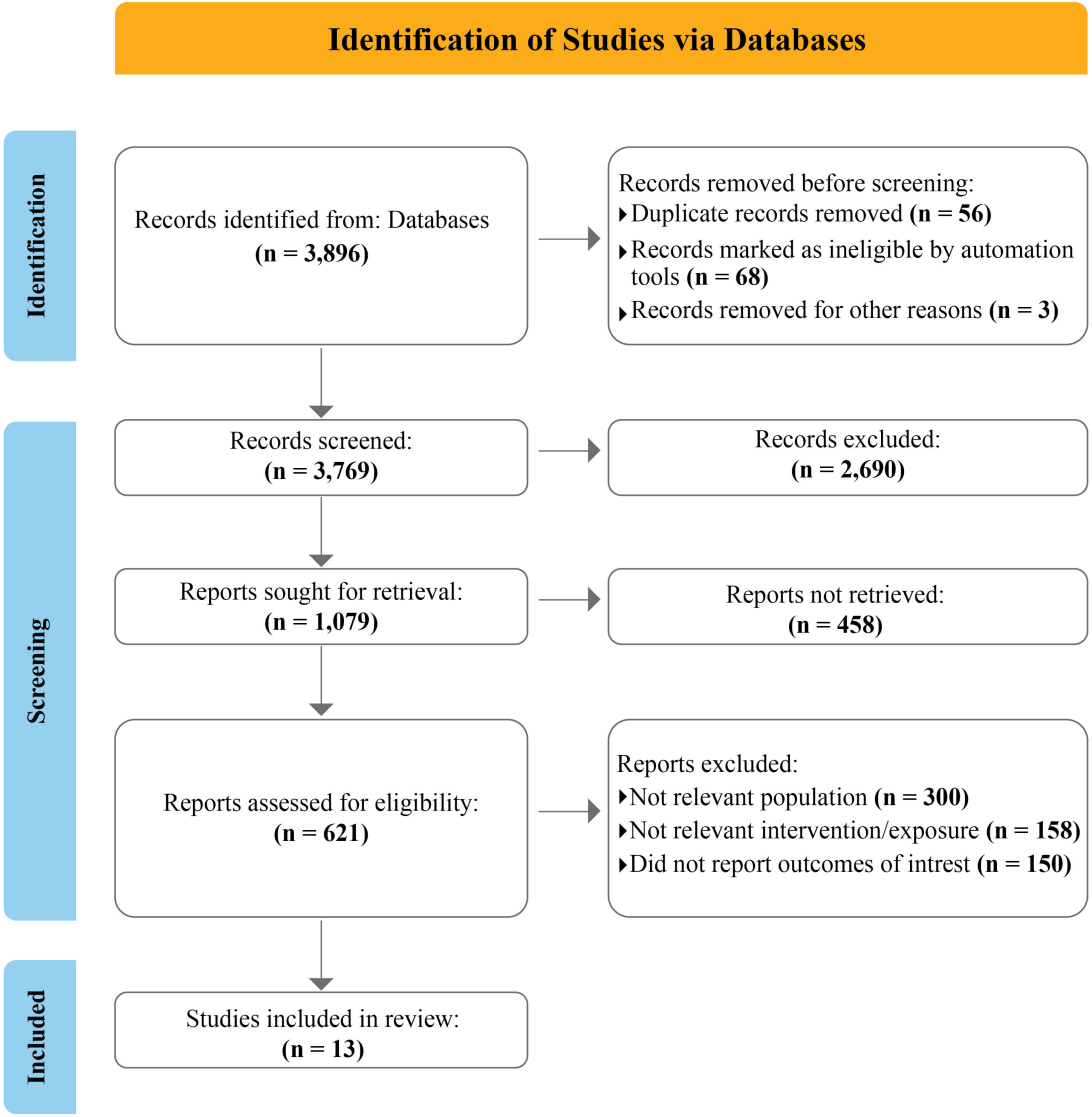
PRISMA flow diagram summarizing study identification and selection.

A database search identified 3,896 records. After duplicate removal and screening, 1,079 records were subjected to title and abstract review, and 621 full texts were assessed for eligibility. Thirteen studies met the inclusion criteria and were included in this qualitative analysis (21–29). Risk of bias was assessed using the Cochrane RoB 2 tool for randomized trials and ROBINS−I for observational studies. The study selection process and reasons for exclusion are shown in the PRISMA flow diagram (**Figure 1**).

### 3.1 Characteristics of included studies

All the included studies were published between 2012 and 2026. From each of the included studies, data were extracted on study design, year of publication, author name, type of intervention, follow-up length, sample size, eligibility, outcomes, and conclusions. The characteristics of all included studies are summarized in **Table 1**.

**Table 1:** Summary of characteristics of included studies.

Author, year	Country	Study design	Study population	Age, BMI	Intervention group details	Co-medication	Comparator group details	Treatment duration (weeks)	N in each arm	Key Findings
Abdul-Ghani et al. (22); 2018	Qatar	Prospective, randomized, open-label study	Patients with poorly controlled, insulin-treated T2DM and patients with poorly controlled non-insulin-treated T2DM	Mean age: 53 ± 2 and Mean BMI: 36.4 ± 1.7 for intervention; Mean age: 51 ± 2 and Mean BMI: 31.2 ± 1.9 for comparator	Dapagliflozin 10 mg plus pioglitazone 30 mg	Insulin	Dapagliflozin	16 weeks	Intervention:18 and Comparator:10	Pioglitazone may be useful as an adjunctive therapy to stop the increase in plasma ketone concentration caused by dapagliflozin
Cho et al. (Initial study) (23); 2024	Korea	Prospective, multicenter, randomized, double-blind, placebo-controlled	Patients with T2DM inadequately controlled with metformin and dapagliflozin	Age: 19 years or older; Mean age: 55.6 years, Mean BMI: 26.5 kg/m2	Pioglitazone 30 mg	Metformin and Dapagliflozin	Placebo	24 weeks	Intervention: 124 and Comparator: 124	HbA1c levels were reduced significantly more in the intervention group than in the placebo group
Cho et al. (Extension study) (23); 2024	Korea	Open-label extension study	Patients with T2DM inadequately controlled with metformin and dapagliflozin	Age: 19 years or older; Mean age: 55.6 years, Mean BMI: 26.5 kg/m2	Pioglitazone 30 mg	Metformin and Dapagliflozin	Placebo	24 weeks	Intervention: 95 and Comparator: 96	The beneficial glycemic impact with the addition of pioglitazone was maintained in this extension study
Gupta et al. (24); 2023	Central India	Randomized trial	Patients with T2DM with HbA1c between 7.0%-10.0% despite being on diet and exercise therapy and metformin 500 mg for at least 3 months	Age≥40 years	Pioglitazone 15 mg and Dapagliflozin 10 mg	Metformin 500 mg and lifestyle modification	Dapagliflozin 10 mg	12 weeks	–	Combination therapy resulted in improved lipid profile, glycemic and blood pressure control. Slight reductions in weight were also observed.
Heo et al. (Initial study) (25); 2024	Korea	Multicenter, double-blind, placebo-controlled, randomized, phase 3 study	Patients with T2DM inadequately controlled with metformin and dapagliflozin	Age: ≥ 19 years; Mean age: 57.2 ± 10.14; BMI: ≤ 45; Mean BMI: 26.3 ± 3.5	Pioglitazone 15 mg	Metformin ≥1,000 mg/day and dapagliflozin 10 mg/day	Placebo	24 weeks	Intervention: 125 and Comparator: 125	Pioglitazone add-on therapy resulted in significant improvements in glycemic control with a favorable safety profile
Heo et al. (Extension study) (25); 2024	Korea	Open-label extension study	Patients with T2DM inadequately controlled with metformin and dapagliflozin	Age: ≥ 19 years; Mean age: 57.2 ± 10.14; BMI: ≤ 45; Mean BMI: 26.3 ± 3.5	Patients in the pioglitazone group continued receiving pioglitazone 15 mg	Metformin ≥1,000 mg/day and dapagliflozin 10 mg/day	Patients in the placebo group in the initial study received pioglitazone	24 weeks	–	Sustained reduction in HbA1C and FBG was observed
Lim et al. (Initial study) (26); 2024	Korea	Multicenter, double-blind, placebo-controlled, randomized, parallel comparison, phase 3 trial	Patients with T2DM suboptimally managed on metformin ≥1000 mg/day plus dapagliflozin 10 mg/day dual therapy for 8 weeks	Age: 57.7 ± 10.0 years for intervention and 58.2 ± 10.1 years for comparator; BMI: 25.8 ± 3.4 kg/m2 for intervention and 25.5 ± 3.0 kg/m2 for comparator	Pioglitazone 15 mg	Metformin: 1383 ± 478 mg in the intervention group and 1402 ± 413 mg in the comparator group, and dapagliflozin 10 mg were maintained from the run-in period to the study end.	Placebo	24 weeks	Intervention: 124 and Comparator: 125	Pioglitazone as an add-on therapy resulted in considerable glycemic control, metabolic benefits, and low hypoglycemia risk
Lim et al. (Extension study) (26); 2024	Korea	Multicenter, double-blind, placebo-controlled, randomized, parallel comparison, phase 3 trial	Patients with T2DM suboptimally managed on metformin ≥1000 mg/day plus dapagliflozin 10 mg/day dual therapy for 8 weeks	Age: 57.7 ± 10.0 years for intervention and 58.2 ± 10.1 years for comparator; BMI: 25.8 ± 3.4 kg/m2 for intervention and 25.5 ± 3.0 kg/m^2^ for comparator	Patients in the pioglitazone group continued receiving pioglitazone 15 mg	Metformin: 1383 ± 478 mg in the intervention group and 1402 ± 413 mg in the comparator group, and dapagliflozin 10 mg were maintained from the run-in period to the study end.	Patients in the placebo group in the initial study received pioglitazone	24 weeks	Intervention: 104 and Comparator: 109	Treatment during this extension study in the placebo group was sufficient to reach the same effects as observed in the pioglitazone group.
Lin et al. (27); 2025	China	Prospective, randomized, controlled, multicenter study	Patients with inadequately controlled T2DM	Age: 18–75 years; Mean BMI for intervention: 25.75 and for comparator: 26.08	Pioglitazone /Metformin fixed dose combination with Dapagliflozin	–	Basal insulin with metformin	16 weeks	Intervention: 73 and Comparator: 74	Patients treated with combination therapy had achieved the target HbA1c without hypoglycemia, with a significant reduction in body weight and improvement in PPG, systolic blood pressure, and lipid profile
Rosenstock et al. (Initial study) (28); 2012	Argentina, Canada, India, Mexico, Peru, the Philippines, Taiwan, and the United States	Randomized, double-blind, placebo-controlled, parallel group	Patients with T2DM inadequately controlled on pioglitazone	Age≥18; BMI ≤ 45.0	Dapagliflozin 10 mg+Pioglitazone≥30 mg	–	Placebo+Pioglitazone≥30 mg	24 weeks	Intervention: 140 and Comparator: 139	Addition of dapagliflozin to pioglitazone reduced HbA1c levels and attenuated pioglitazone-related weight gain without increasing the risk of hypoglycemia
Rosenstock et al. (Extension study) (28); 2012	Argentina, Canada, India, Mexico, Peru, the Philippines, Taiwan, and the United States	Randomized, double-blind, placebo-controlled, parallel group	Patients with T2DM inadequately controlled on pioglitazone	Age≥18; BMI ≤ 45.0	Dapagliflozin 10 mg+Pioglitazone≥30 mg	–	Placebo+Pioglitazone≥30 mg	24 weeks	Intervention: 29 and Comparator: 95	Sustained glycemic benefits were observed with this combination.
Seshadri et al. (29); 2025	India	Retrospective study	Patients with T2DM and MASLD who were prescribed pioglitazone and/or an SGLT2i	Age: 53.7 ± 13.4; BMI: 25.2 ± 9.9 kg/m2	Pioglitazone and SGLT2i	–	SGLT2i	> 12 weeks	Intervention: 29 and Comparator: 140	Combination therapy of pioglitazone and SGLT2i resulted in a significant reduction of the FIB-4 index and APRI score
Singh, et al. (21); 2026	India	Multicenter, randomized, open label, active-controlled, phase 3 trial	Adults with T2DM inadequately controlled on metformin	Adults ≥18 years; BMI ≤45 kg/m²	Fixed-dose combination of dapagliflozin 10 mg plus pioglitazone 15 mg	–	Loose combination of dapagliflozin 10 mg and pioglitazone 15 mg	12 weeks	Intervention: 85; Comparator: 84	The fixed-dose dapagliflozin–pioglitazone combination demonstrated non-inferior glycemic efficacy compared with the loose combination, with comparable safety and tolerability, and favorable effects on body weight and metabolic parameters.

APRI, aspartate aminotransferase to platelet ratio index; BMI, body mass index; FBG, fasting blood glucose; FIB‐4, fibrosis‐4 Index; HbA1c, glycated hemoglobin; MASLD, metabolic dysfunction‐associated steatotic liver disease; N, number of patients; PPG, post‐prandial blood glucose; SGLT2, sodium glucose co‐transporter 2; T2DM, type 2 diabetes mellitus.

### 3.2 Primary outcomes

The primary outcomes of this meta-analysis were glycemic parameters including HbA1c, FBG, and PPG. Exploratory pooled-arm estimates contributed only to glycemic outcomes and did not influence hepatic or safety synthesis.

#### 3.2.1 HbA1c

**Figure 2:**
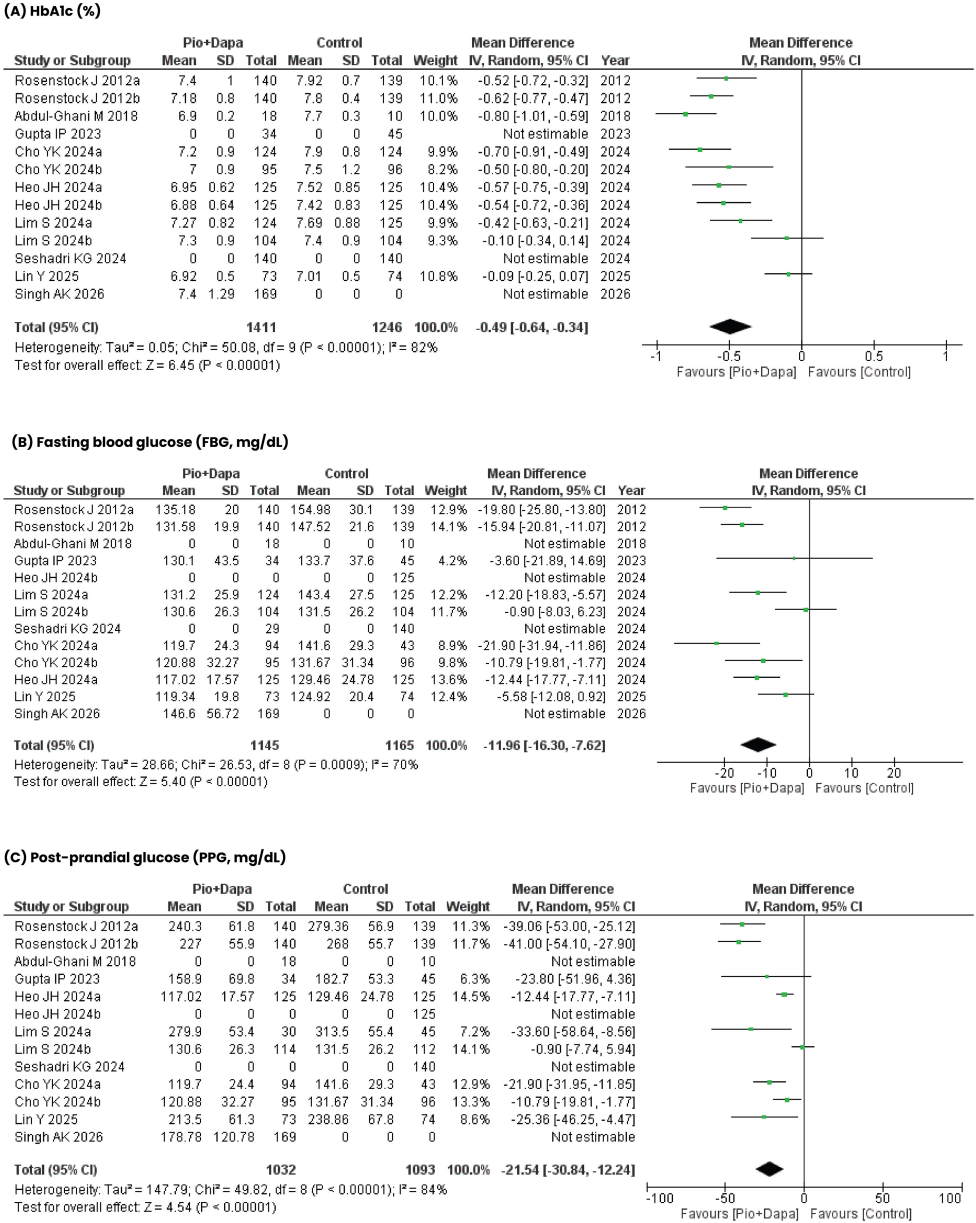
Forest plots for glycemic outcomes with dapagliflozin plus pioglitazone versus comparators (placebo, standard care, or active therapy) using a random-effects model: **(A)** HbA1c (%), **(B)** FBG (mg/dL), and **(C)** PPG (mg/dL). Values are mean differences (MD) with 95% confidence intervals (CI); diamonds represent pooled effects. Negative MD indicates improvement. Corresponding funnel plots are shown in **Supplementary Figures S1** (HbA1c), **S2** (FBG), and **S3** (PPG).

Ten studies evaluated the effects of pioglitazone combined with dapagliflozin on HbA1c levels. Pooled analysis showed a significant reduction in HbA1c with combination therapy, with a mean difference of −0.49% (95% confidence interval [CI] −0.64 to −0.34) and substantial heterogeneity (I² = 82%) (**Figure 2A**).

#### 3.2.2 Fasting blood glucose

Nine studies reported changes in FBG with pioglitazone combined with dapagliflozin. The pooled analysis demonstrated a significant reduction in FBG levels, with a mean difference of −11.96 mg/dL (95% CI −16.30 to −7.62), accompanied by substantial heterogeneity (I² = 70%) (**Figure 2B**).

#### 3.2.3 Post-prandial glucose

Nine studies evaluated the changes in PPG levels with pioglitazone combined with dapagliflozin (23–28). The pooled analysis demonstrated a significant reduction in PPG, with a mean difference of −21.54 mg/dL (95% CI −30.84 to −12.24), accompanied by considerable heterogeneity (I² = 84%) (**Figure 2C**). Funnel plots for glycemic outcomes are presented in **Supplementary Figures S1**-**S3.** Visual inspection did not reveal marked asymmetry; however, the interpretation was limited by the small number of studies contributing to each analysis.

### 3.3 Secondary outcomes

Secondary outcomes included anthropometric, metabolic, hepatic, and safety parameters associated with dapagliflozin plus pioglitazone therapy.

#### 3.3.1 Aspartate aminotransferase

**Figure 3:**
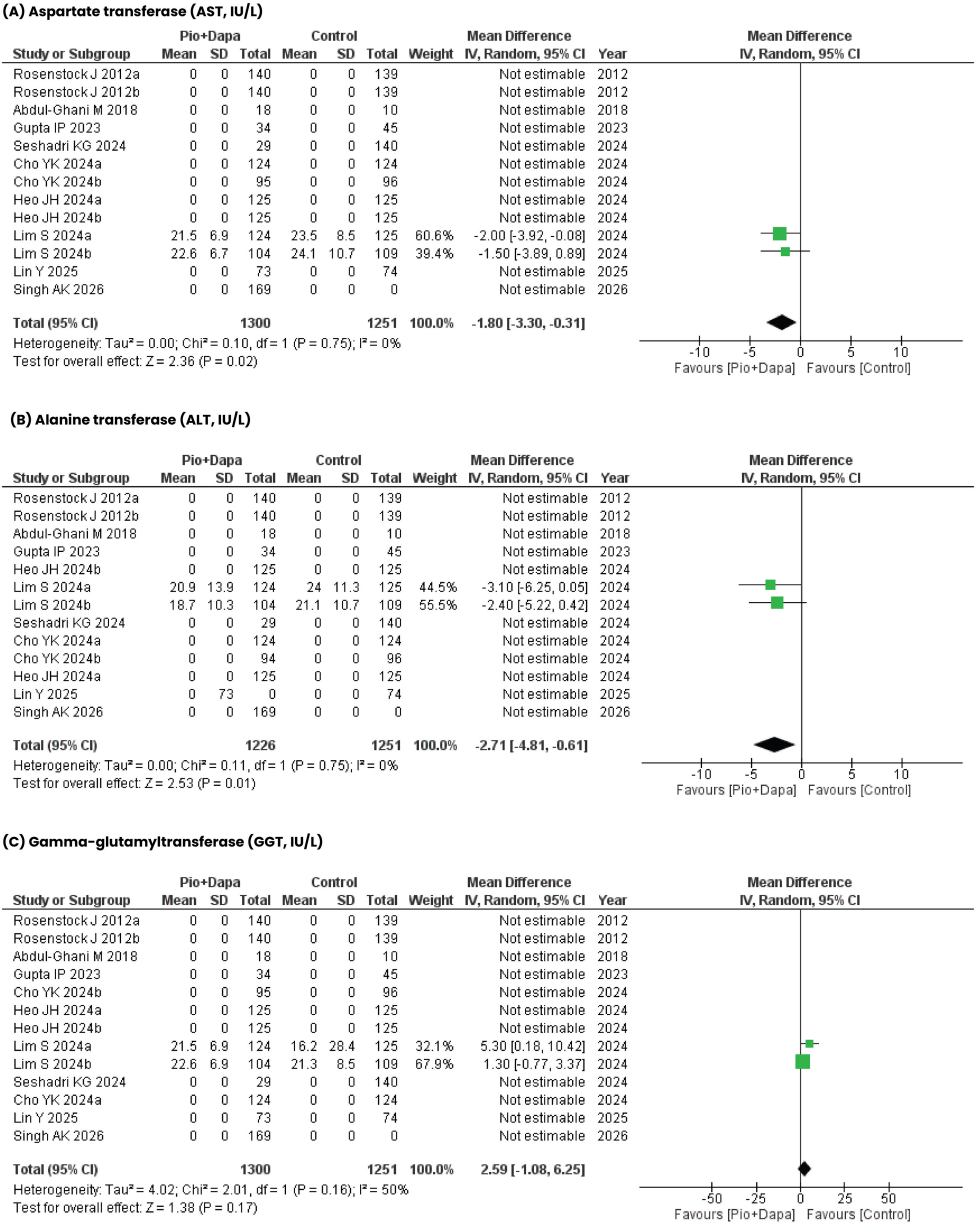
Forest plots for hepatic enzyme outcomes with dapagliflozin plus pioglitazone versus comparators (placebo, standard care, or active therapy) using a random-effects model: **(A)** aspartate aminotransferase (AST, U/L), **(B)** alanine aminotransferase (ALT, U/L), and **(C)** gamma-glutamyl transferase (GGT, U/L). Values are mean differences (MD) with 95% confidence intervals (CI); diamonds represent pooled effects.

Two studies reported changes in AST levels with pioglitazone combined with dapagliflozin. The pooled analysis demonstrated a small but statistically significant reduction in AST, with a mean difference of −1.80 U/L (95% CI −3.30 to −0.31), and no observed heterogeneity (I² = 0%) (**Figure 3A**).

#### 3.3.2 Alanine aminotransferase

Two studies reported changes in ALT with pioglitazone combined with dapagliflozin. The pooled analysis demonstrated a modest but statistically significant reduction in ALT, with a mean difference of −2.71 U/L (95% CI −4.81 to −0.61), and no observed heterogeneity (I² = 0%) (**Figure 3B**).

#### 3.3.3 Gamma-glutamyl transferase

Two studies have reported changes in GGT levels with pioglitazone combined with dapagliflozin. The pooled analysis did not demonstrate a statistically significant effect on GGT levels, with a mean difference of -2.59 U/L (95% CI −1.08 to 6.25) and moderate heterogeneity (I² = 50%) (**Figure 3C**). Corresponding Funnel plots were generated for AST, ALT and GGT -**Supplementary Figures S4**–**S6**.

#### 3.3.4 Non-invasive MASLD indices and fibrosis surrogates

Across studies reporting hepatic endpoints not amenable to meta-analysis, dual pioglitazone–dapagliflozin therapy was associated with consistent improvements in non-invasive markers of steatosis and fibrosis. In longitudinal cohorts, fibrosis surrogates declined with combination therapy, including reductions in FIB-4 (1.92 to 1.68) and APRI (0.41 to 0.33) over one year (29). Trials incorporating steatosis metrics demonstrated improvements in validated indices, including reductions in NAFLD Liver Fat Score with pioglitazone add-on therapy (27) and significant decreases in Fatty Liver Index following dapagliflozin initiation.

#### 3.3.5 Body weight

**Figure 4:**
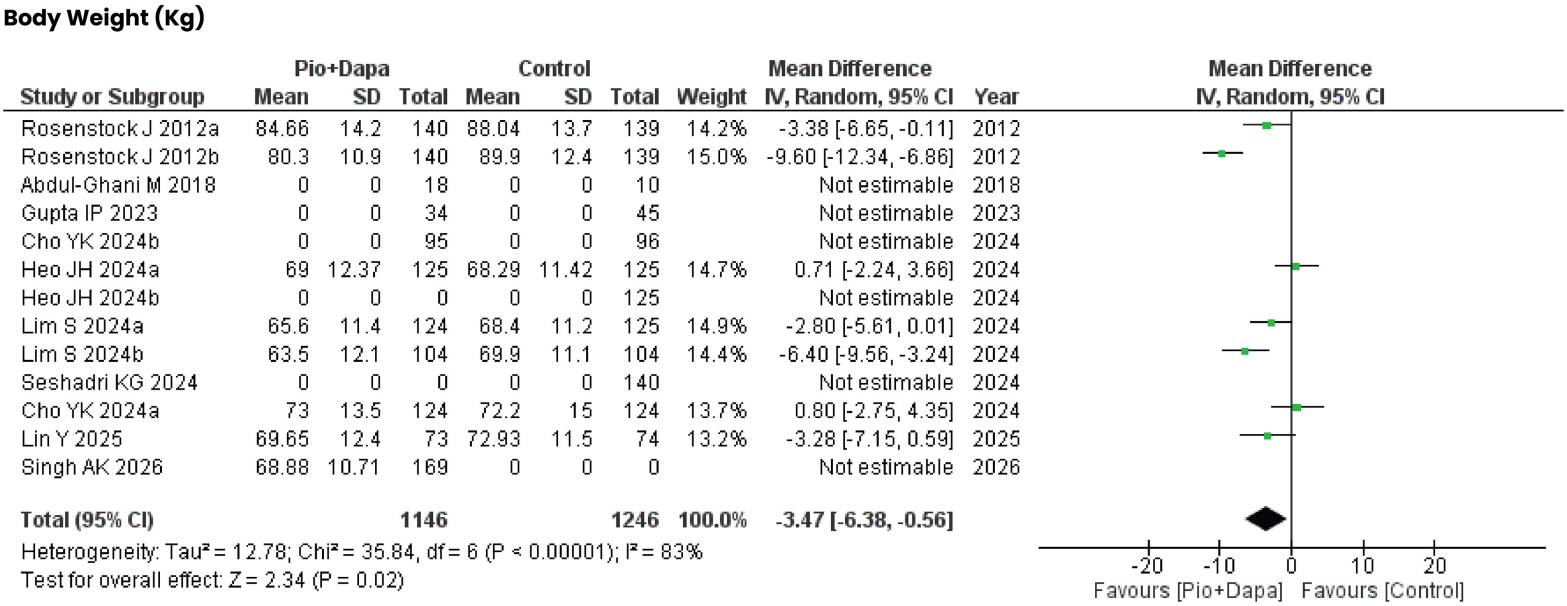
Forest plot showing the effect of dapagliflozin plus pioglitazone versus comparators (placebo, standard care, or active therapy) on body weight using a random-effects model. Values are mean differences (MD) in kilograms (kg) with 95% confidence intervals (CI). The diamond represents the pooled effect estimate; negative values indicate greater body weight reduction with combination therapy.

Seven studies reported changes in body weight with dapagliflozin plus pioglitazone compared with the control. Of these, seven contributed estimable data to the meta-analysis. Pooled analysis demonstrated a statistically significant reduction in body weight, with a mean difference of −3.47 kg (95% CI −6.38 to −0.56), accompanied by considerable heterogeneity (I² = 83%) (**Figure 4**). The corresponding funnel plot is shown in **Supplementary Figure S7**.

#### 3.3.6 Lipid outcomes

**Figure 5:**
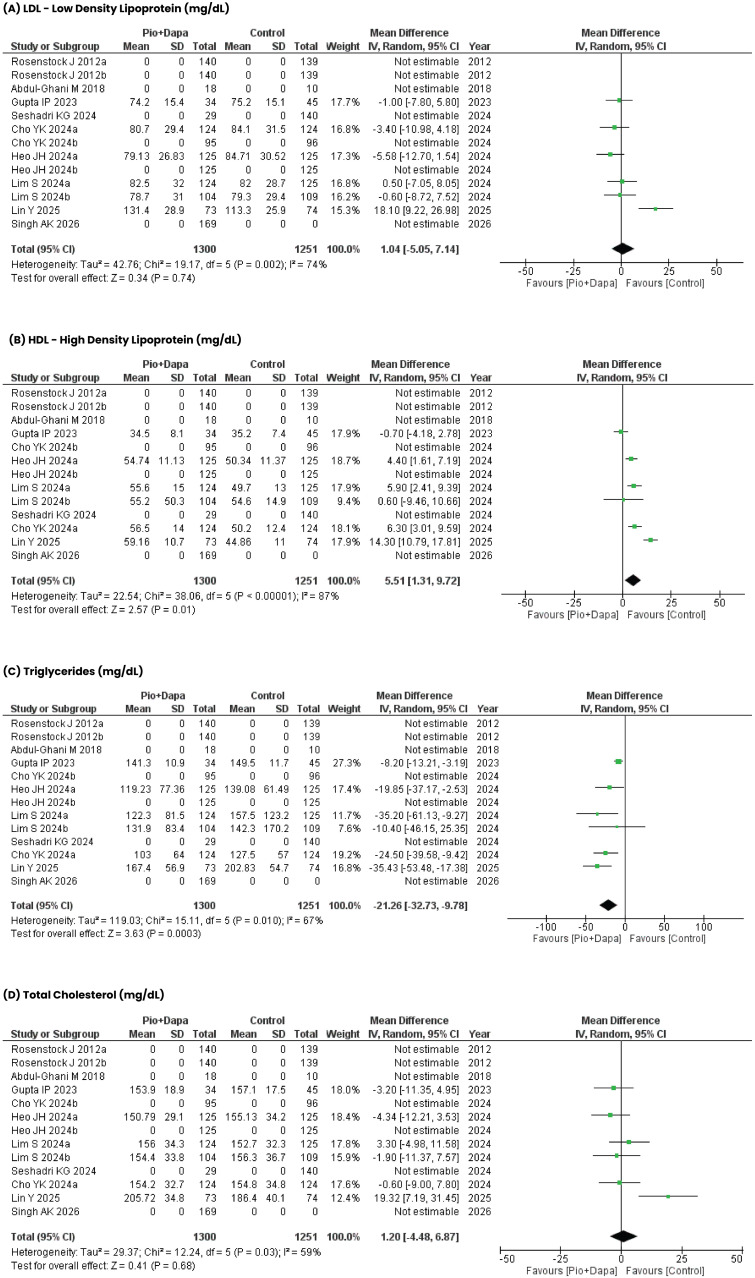
Forest plots of lipid outcomes with dapagliflozin plus pioglitazone versus comparators (placebo, standard care, or active therapy) using an inverse-variance random-effects model: **(A)** LDL cholesterol (mg/dL), **(B)** HDL cholesterol (mg/dL), **(C)** triglycerides (mg/dL), and **(D)** total cholesterol (mg/dL). Squares represent study-specific effect estimates and diamonds represent pooled effects; horizontal lines indicate 95% confidence intervals.

Six studies assessed lipid parameters with dapagliflozin plus pioglitazone compared with control. The pooled analysis demonstrated a significant increase in high-density lipoprotein (HDL) cholesterol, with a mean difference (MD) of 5.51 mg/dL (95% confidence interval [CI] 1.31 to 9.72), and a significant reduction in triglyceride levels, with an MD of −21.26 mg/dL (95% CI −32.73 to −9.78). In contrast, no statistically significant effects were observed for low-density lipoprotein (LDL) cholesterol (MD -1.04 mg/dL, 95% CI −5.05 to 7.14) or total cholesterol (MD -1.20 mg/dL, 95% CI −4.48 to 6.87). Heterogeneity ranged from moderate to high across lipid outcomes (**Figure 5**). Corresponding funnel plots were generated for lipid outcomes (**Supplementary Figures S8**–**S11**).

#### 3.3.7 HOMA-IR and HOMA-B

Four studies reported the effects of insulin resistance and β-cell function on homeostatic indices. In a multicenter, placebo-controlled trial conducted on a metformin-based dapagliflozin backbone, the addition of a TZD resulted in significantly lower HOMA-IR and preservation of HOMA-B at 24 weeks compared with placebo, with a dose–response relationship favoring a higher TZD dose. Three additional studies reported concordant directional findings, showing reductions in HOMA-IR and stable or improved HOMA-B with Dapagliflozin-pioglitazone combinations compared to control regimens, although formal between-group statistical comparisons were not consistently reported.

#### 3.3.8 Safety outcomes

Across the included studies, the overall incidence of adverse events (AEs) was generally comparable between dapagliflozin-pioglitazone combination therapy and comparator groups. In the trials, treatment-emergent AE rates ranged from 20.6-31.5% in combination arms versus 20.8-29.6% in comparator arms, with no statistically significant between-group differences reported. Serious adverse events were rare, occurring in 0-4.3% of combination therapy recipients versus 0–2.9% in comparators, and no study demonstrated a statistically significant excess risk attributable to the combination. No deaths were attributed to combination therapy, and treatment discontinuations due to AEs were low across all studies (0-3.6%). Hypoglycemia was consistently minimal; no major hypoglycemic episodes were reported in any randomized trial, and in one active-comparator study, hypoglycemia was significantly lower with the oral combination (2.74%) than with insulin-based therapy (13.51%). Peripheral edema, a recognized concern with pioglitazone monotherapy, was attenuated by concurrent dapagliflozin, with edema rates of 0.8-4.3% across combination arms. Genital mycotic infections, consistent with the SGLT2i class effect, were reported in up to 9.2% of dapagliflozin-treated patients versus 2.9% with placebo, and were uniformly mild to moderate with no treatment withdrawals. The reported adverse drug reactions were consistent with the known class-specific safety profiles of SGLT2i and pioglitazone, and no new or unexpected safety signals emerged from pooled randomized or observational data.

## 4 Discussion

### 4.1 Integrated mechanisms underpinning glycemic and hepatic benefits

Dapagliflozin and pioglitazone may provide complementary metabolic effects in adults with T2DM and MASLD (6, 24, 30–32). Dapagliflozin promotes glucosuria, modest caloric loss, and reductions in adiposity, while pioglitazone improves peripheral insulin sensitivity, lowers free fatty acid flux, and increases adiponectin through PPARγ activation (24, 30–32). Together, these mechanisms provide a plausible biological explanation for the observed improvements in glycemic outcomes, body weight profile, liver enzymes, and non-invasive markers of hepatic steatosis across the included studies (6, 28, 33). However, these mechanistic interpretations should be viewed as supportive context rather than direct evidence of disease modification, as the current hepatic evidence is based largely on surrogate or non-invasive markers, with limited biopsy-confirmed data on steatohepatitis or fibrosis.

### 4.2 Glycemic efficacy and metabolic effects of dapagliflozin–pioglitazone

Across 13 studies, dapagliflozin–pioglitazone combination therapy was associated with significant reductions in HbA1c, fasting plasma glucose, and post-prandial glucose. The pooled HbA1c reduction of approximately −0.49% is consistent with an additive glycemic effect when SGLT2 inhibition is combined with pioglitazone-based insulin sensitization (31, 34–37). Improvements in insulin resistance indices and β-cell function further support the metabolic benefit of dual therapy (38–40). However, given the substantial heterogeneity observed for several glycemic outcomes, these estimates should be interpreted as average effects across clinically diverse study populations rather than as a uniform treatment response.

### 4.3 Attenuation of pioglitazone-associated weight gain by dapagliflozin

A clinically relevant finding of this meta-analysis was the attenuation of pioglitazone-associated weight gain with concurrent dapagliflozin. Although pioglitazone monotherapy is commonly associated with weight gain through fluid retention and adipose tissue expansion, dapagliflozin promotes caloric loss through glucosuria and may reduce visceral adiposity (28, 41). In our pooled analysis, combination therapy was associated with significantly less weight gain than pioglitazone alone (MD −3.47 kg, 95% CI −6.38 to −0.56). This weight-neutral or weight-attenuating profile may improve treatment acceptability while preserving the insulin-sensitizing benefit of pioglitazone. Longer-duration trials are needed to determine whether these metabolic effects translate into durable hepatic or histologic benefit (15, 17).

### 4.4 Comparison with prior meta-analyses and positioning of current evidence

Our findings align with and extend prior meta-analyses evaluating the SGLT2i–pioglitazone combination therapy. Liao et al. reported improvements in glycemic control and liver fat with dual therapy, but included a limited number of trials and did not assess histologic outcomes (38). Anson et al. demonstrated favorable metabolic effects of pioglitazone combined with SGLT2is or GLP-1 receptor agonists; however, the available data were insufficient to evaluate steatohepatitis resolution (42). In an Asian population, Liu et al. observed comparable reductions in hepatic steatosis with pioglitazone and SGLT2i monotherapy, along with greater weight gain with pioglitazone alone (43). In contrast, the present meta-analysis synthesizes evidence from a larger and more diverse body of randomized and real-world studies and provides a unified assessment across glycemic, hepatic, histologic, and weight-related domains. Collectively, these findings support a potential complementary role for combined SGLT2i and TZD therapy in adults with T2DM and MASLD, particularly when insulin resistance, hyperglycemia, and ectopic fat accumulation coexist.

### 4.5 Alignment with current diabetes and MASLD clinical guidelines

The current American Diabetes Association (ADA) Standards of Care prioritize organ-protective therapies in T2DM, recommending SGLT2is for patients with cardiovascular or kidney disease and supporting their use in high cardiometabolic risk profiles (15). AASLD and EASL–EASD–EASO guidelines conditionally recommend pioglitazone for biopsy-proven NASH, citing evidence for histologic improvement while noting concerns related to weight gain, fluid retention, and patient selection (14, 15). SGLT2is are not currently guideline-endorsed as specific MASLD/MASH therapies, although they have established cardiovascular and renal benefits in T2DM and emerging evidence for hepatic fat reduction (12, 44). In this context, the present findings position dapagliflozin–pioglitazone as a potentially useful oral combination for selected patients with T2DM and MASLD, particularly where weight gain from pioglitazone is a concern. However, because hepatic outcomes were largely based on non-invasive markers, this combination should not be interpreted as a guideline-endorsed treatment for MASH or fibrosis.

### 4.6 Sources of heterogeneity and population-specific effects

The between-study heterogeneity varied across outcomes and warrants explicit consideration in interpreting pooled glycemic and body weight estimates. For HbA1c (I² = 82%), FBG (I² = 70%), PPG (I² = 84%), and body weight (I² = 83%), several factors likely contribute to this variability. First, study design heterogeneity, including the mix of randomized controlled trials and observational studies, may introduce differential treatment effect estimates, as observational cohorts are subject to confounding by indication and variable outcome ascertainment. Second, treatment duration ranged from 12 to 52 weeks across included studies, and shorter-duration trials may capture earlier glycemic responses without the full metabolic adaptation seen with longer follow-up. Third, baseline HbA1c varied considerably across study populations; patients with higher baseline HbA1c typically demonstrate greater absolute reductions, inflating pooled estimates relative to studies enrolling better-controlled populations. Fourth, background therapies differed substantially across trials, with some studies conducted on a metformin-dapagliflozin backbone, others on insulin, and one on a basal insulin-metformin comparator, introducing pharmacodynamic interactions that may modify the incremental benefit of pioglitazone add-on therapy. Fifth, pioglitazone doses varied across studies (15 mg vs. 30 mg), and dose-dependent effects on insulin sensitivity and body weight may further contribute to between-study variability. Minimal heterogeneity was observed for transaminase endpoints (ALT and AST; I² = 0%), whereas moderate heterogeneity was noted for GGT (I² = 50%), likely reflecting differences in baseline liver disease severity and metabolic profiles. The random-effects model was used throughout to account for this variability, and findings should be interpreted as reflecting the average effect across a heterogeneous clinical landscape rather than a uniform treatment response. These observations highlight the need for adequately powered trials with predefined stratification by baseline HbA1c, fibrosis stage, and background therapy to better characterize the sources of heterogeneity (14, 32).

### 4.7 Safety profile and implications for patient selection

Across the included studies, adverse event rates were comparable between dapagliflozin–pioglitazone combination therapy and control regimens (RR 1.02, 95% CI 0.94 to 1.11), with serious adverse events remaining rare and evenly distributed (38). In the PRO-1 trial, mild adverse events occurred in approximately 5% of participants, with no serious events or hypoglycemia reported (45). No new hepatic, renal, or cardiovascular safety signals were identified across pooled randomized and observational studies. Weight gain and edema typically associated with TZD therapy were attenuated by concurrent SGLT2 inhibition, with the present meta-analysis demonstrating significantly less weight gain versus pioglitazone alone (MD −3.47 kg, 95% CI −6.38 to −0.56). This effect likely reflects SGLT2i-mediated caloric loss, osmotic diuresis, and natriuresis, partially offsetting pioglitazone-related fluid retention and adipose redistribution (24, 41). The risk of hypoglycemia remained minimal, with no severe episodes reported in the dual-therapy arms (38). Genital mycotic infections occurred in less than 5% of patients and were generally mild, manageable, and rarely led to treatment discontinuation (42, 44, 46). Overall, the available safety data support the real-world feasibility of dapagliflozin–pioglitazone combination therapy in appropriately selected adults with T2DM, with or without MASLD, while recognizing the need for longer-term safety follow-up.

### 4.8 Clinical implications and monitoring recommendations

Dapagliflozin–pioglitazone may be considered for selected adults with T2DM, with or without MASLD, who require improved glycemic control and in whom pioglitazone-associated weight gain is a concern. In clinical practice, monitoring should include glycemic response, body weight, edema or volume status, renal function, bone health in higher-risk patients, and symptoms of genital mycotic infection (41–44, 47). For patients with MASLD, liver fat and fibrosis risk may be followed using non-invasive tools such as transient elastography with CAP, MRI-PDFF where available, and serum-based indices such as FIB-4 or the NAFLD fibrosis score (44, 48). Given the limited biopsy-confirmed evidence, treatment decisions should remain individualized and should not rely on presumed histologic benefit.

### 4.9 Limitations and GRADE certainty assessment

The inclusion of both randomized and observational studies introduces inherent uncertainty; observational cohorts are susceptible to confounding by indication and selection bias, which may bias effect estimates in either direction. Although sensitivity analyses restricted to RCTs were not feasible given the small number of contributing studies per outcome, the directional consistency between randomized and observational estimates provides some reassurance regarding robustness. Interpretation of the findings is limited by sparse biopsy data, with only four of 13 studies including histologic assessment, short follow-up (median 24 weeks; range 12–52 weeks), and inclusion of observational cohorts, all of which reduce confidence in histologic endpoints (14, 29, 32, 38). Most trials relied on non-invasive measures such as MRI-PDFF, CAP, and serum based indices including FIB−4 and the NAFLD fibrosis score, which correlate imperfectly with steatohepatitis activity and fibrosis stage (41). In addition, study populations were predominantly Asian, limiting generalizability to other ethnic and geographic groups (14). Funnel plots were generated for all outcomes but have low statistical power to detect asymmetry when fewer than 10 studies contribute to an analysis. Visual inspection did not reveal marked asymmetry; however, given the small number of contributing studies, the absence of detected asymmetry should not be interpreted as evidence against publication bias. Small-study effects cannot be reliably excluded, and the possibility that smaller studies with null or negative findings remain unpublished may have influenced pooled estimates. Overall certainty of evidence, assessed using GRADE, was moderate for glycemic and steatosis outcomes and low for histologic outcomes, as summarized in **Table A1**. These limitations highlight the need for adequately powered, biopsy confirmed randomized trials with follow up of at least 48 weeks to establish effects on steatohepatitis and fibrosis. Until such evidence is available, dual SGLT2is and TZD therapy is best supported for metabolic control and steatosis improvement in populations with overlapping T2DM and MASLD (18, 19).

### 4.10 Future research directions

Future trials should enroll adults with T2DM, including biopsy-confirmed NASH subsets where feasible, and extend the follow-up beyond 96 weeks to assess durability. Head-to-head comparisons of SGLT2i-TZD combinations versus GLP−1 receptor agonist–based regimens (alone and in combination) are needed, with endpoints spanning fibrosis regression, quantitative liver fat by MRI−PDFF, and long-term cardiometabolic outcomes (MACE, renal decline), alongside patient-reported quality of life. Stratified analyses by baseline fibrosis stage, diabetes duration, and ethnicity should identify responder phenotypes and refine the selection. Mechanistic sub-studies (adiponectin, inflammatory markers, and lipidomics) can clarify the pathways underlying the heterogeneity of response. In parallel, real-world registries linking metabolic control to liver outcomes (progression, decompensation, and mortality) will complement randomized controlled trials and inform their safety. Integrated programs comparing and sequencing oral combinations and incretin−based strategies will define how best to deploy combination therapies for comprehensive cardiometabolic care in diverse populations building on emerging real-world and contemporary MASLD evidence (49, 50).

## 5 Conclusion

This systematic review and meta-analysis demonstrated that dapagliflozin–pioglitazone combination therapy was associated with improvements in glycemic control and noninvasive markers of hepatic steatosis in adults with T2DM, with or without MASLD, while maintaining a favorable safety and weight profile. These effects are biologically plausible, reflecting the complementary actions of dapagliflozin-mediated metabolic unloading and pioglitazone-driven insulin sensitization and support a rational dual-pathway approach to address concurrent hepatic and cardiometabolic risk. The certainty of evidence was moderate for metabolic and steatosis outcomes and low for histologic endpoints, reflecting imprecision and indirectness arising from the limited biopsy-confirmed data. Adequately powered randomized trials incorporating histologic endpoints, longer follow-up, and evaluation of cardiovascular and renal outcomes are required to define the long-term disease-modifying effects. Until such evidence is available, dapagliflozin–pioglitazone represents a guideline-consistent, mechanistically grounded option for carefully selected patients with T2DM with or without MASLD.

## Data Availability

The original contributions presented in the study are included in the article/**Supplementary Material**. Further inquiries can be directed to the corresponding author.
