# Supplementary material for: Dapagliflozin & pioglitazone combination therapy in T2DM with or without MASLD - a systematic review and meta-analysis: PRO-2 study
Source: Front Clin Diabetes Healthc. 2026 Jul 20;7:1733995. doi: 10.3389/fcdhc.2026.1733995 (PMC13429781; doi:10.3389/fcdhc.2026.1733995)
Supplement: Supplementary file 1 [file DataSheet1.pdf]

Supplementary Figures

Figure S1. HbA1c: funnel plot analysis

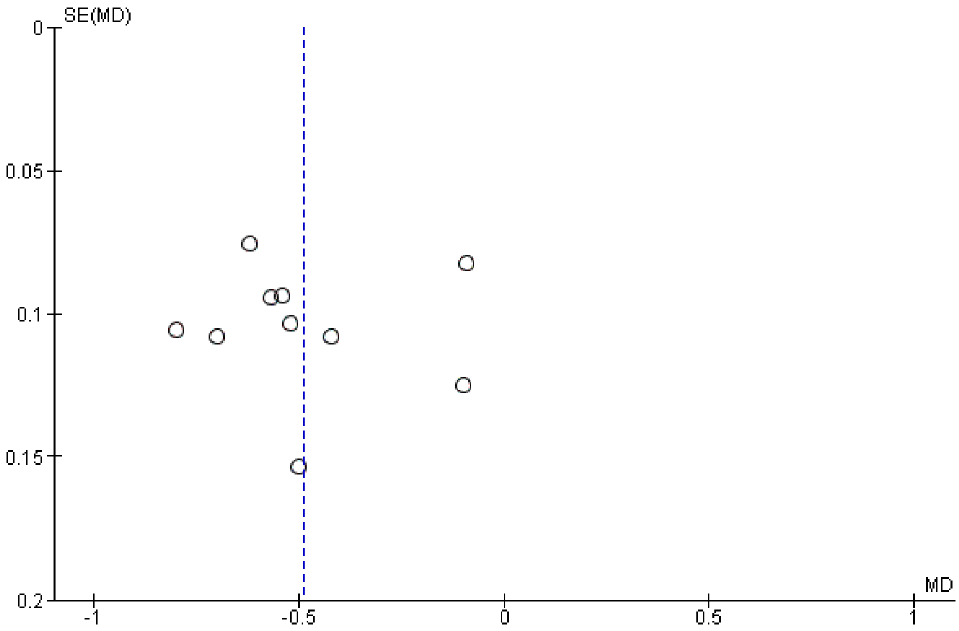

Figure S2. Fasting blood glucose: funnel plot analysis

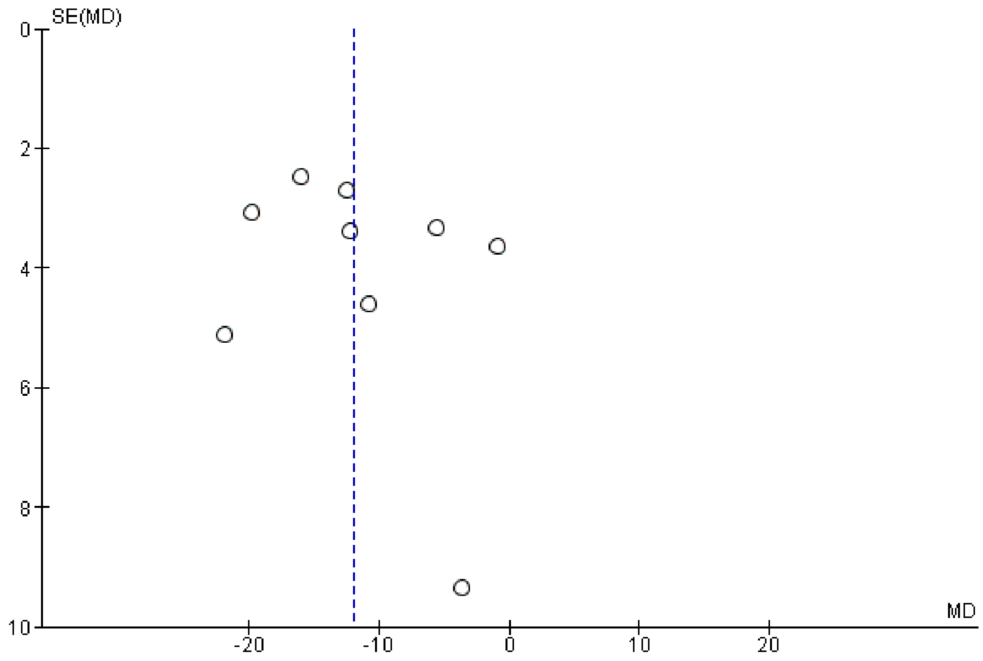

Figure S3. Post-prandial blood glucose: funnel plot analysis

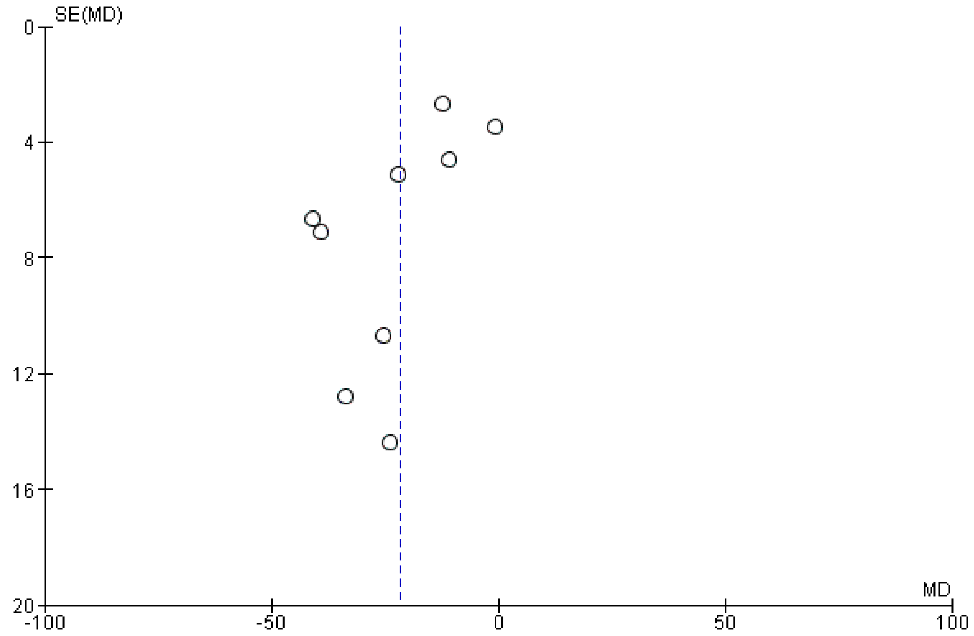

Figure S4. AST: funnel plot analysis

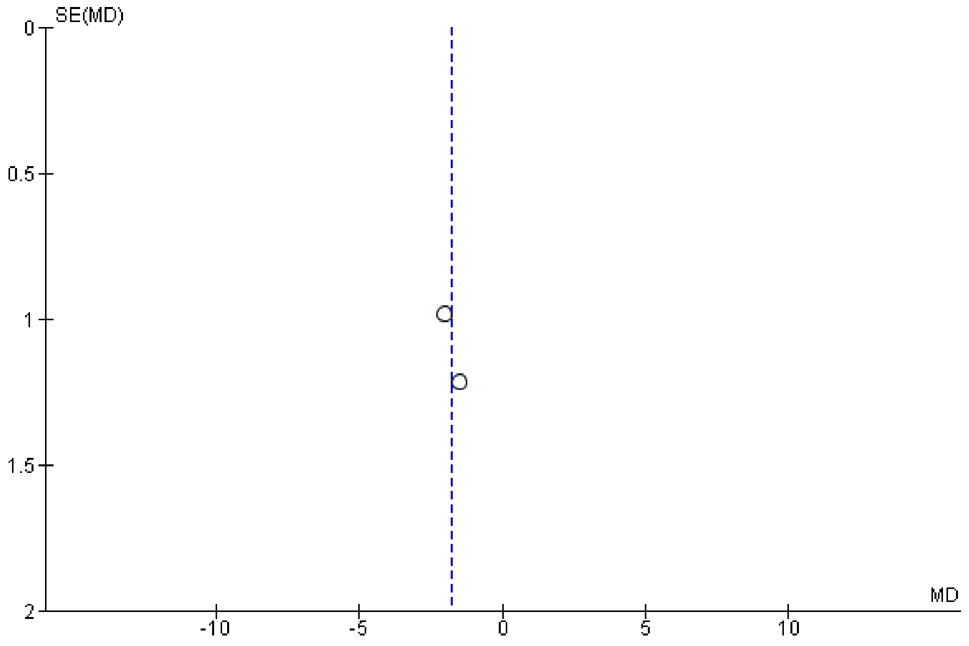

Figure S5. ALT: funnel plot analysis

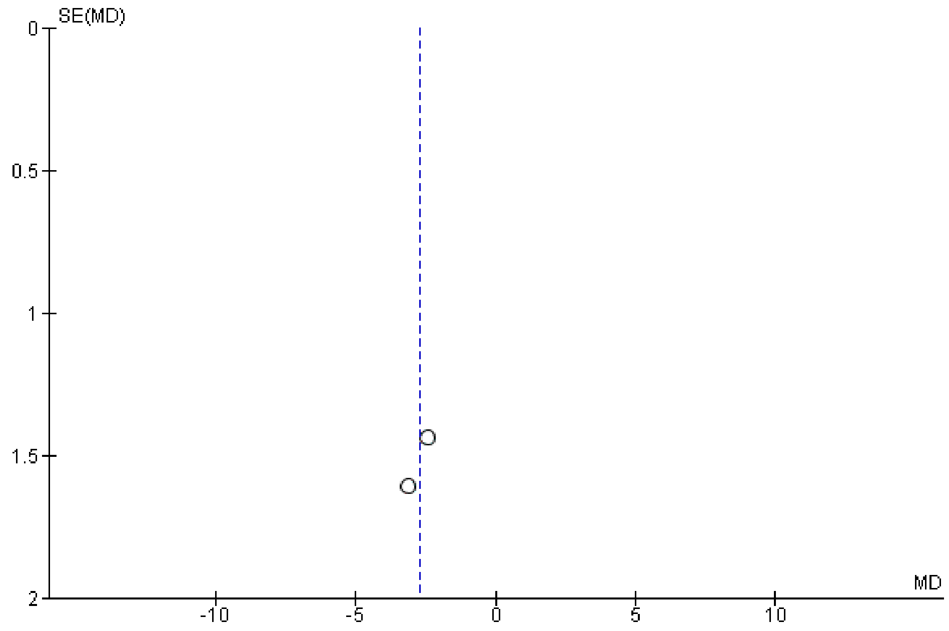

Figure S6. GGT: funnel plot analysis

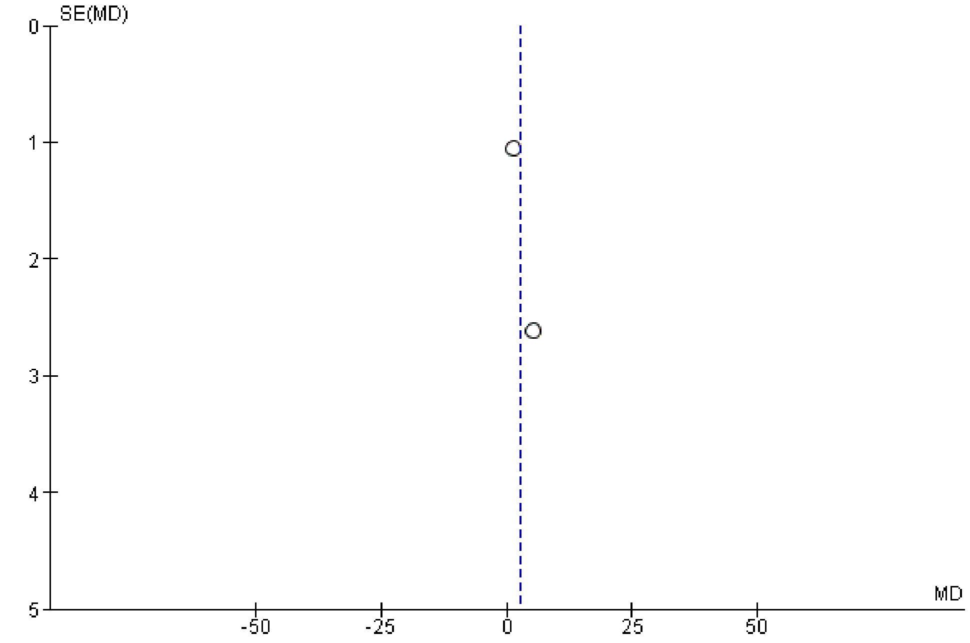

Figure S7. Body Weight: funnel plot analysis

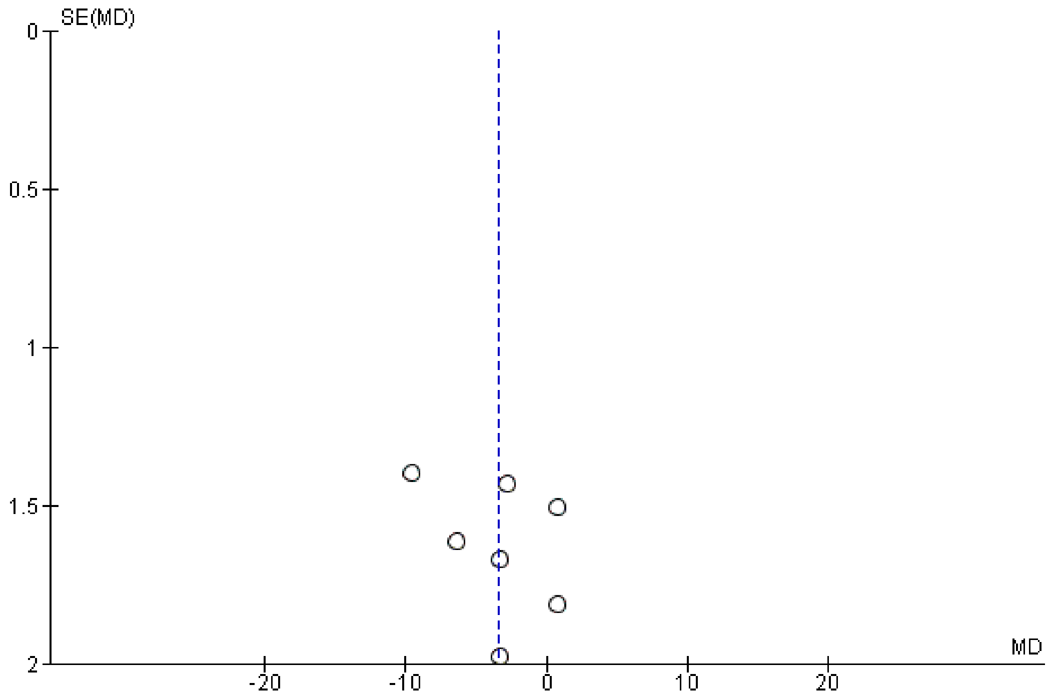

Figure S8. LDL: funnel plot analysis

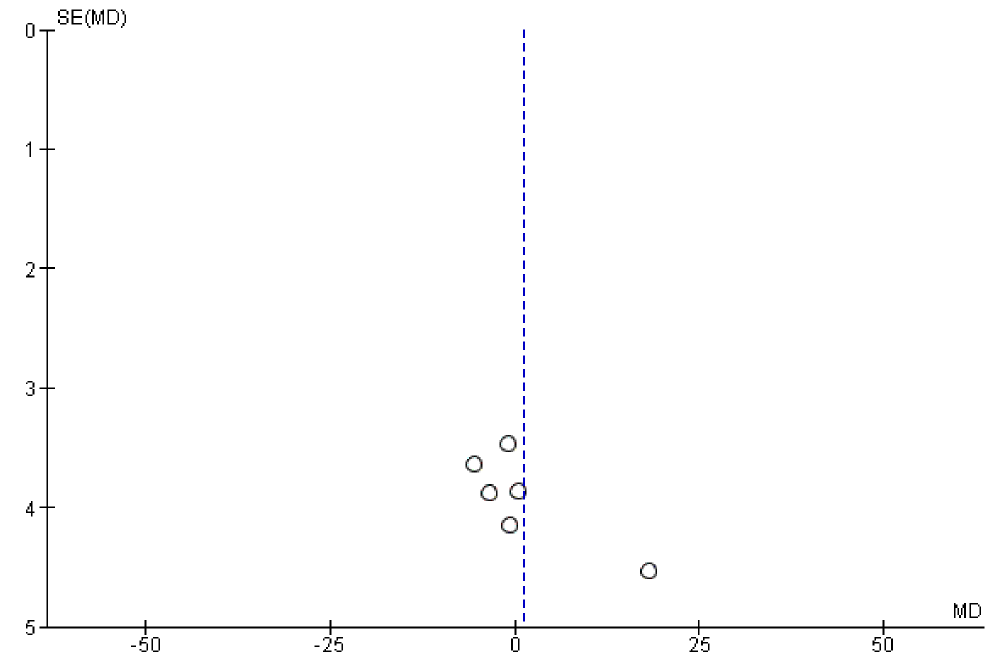

Figure S9. HDL: funnel plot analysis

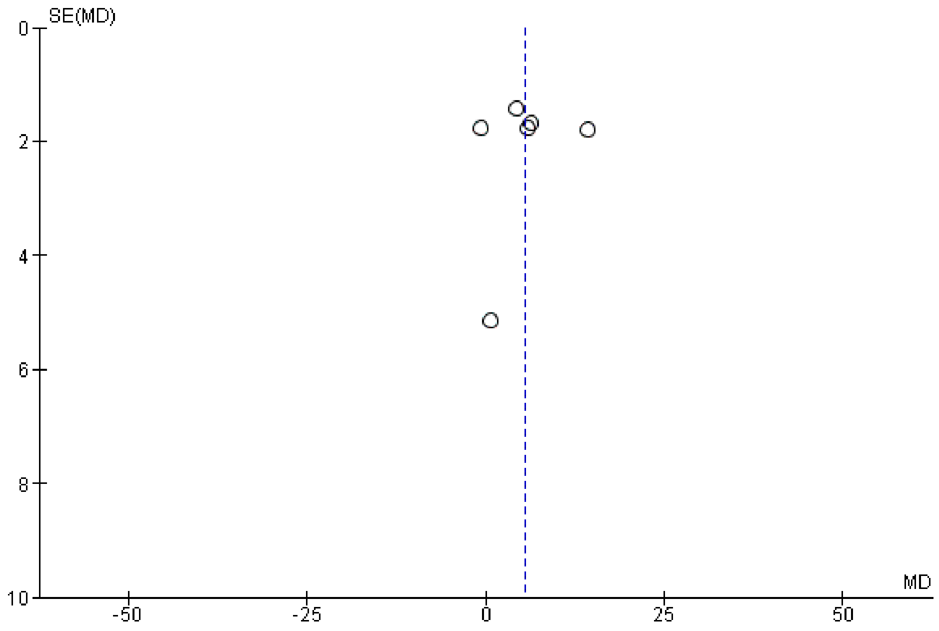

Figure S10.Triglycerides: funnel plot analysis

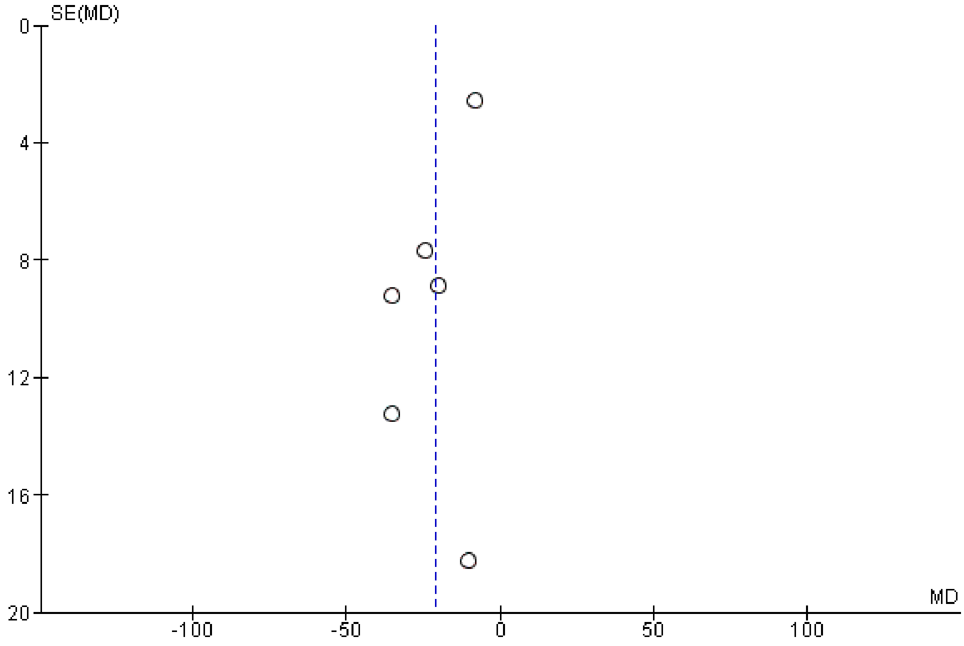

Figure S11.Total Cholesterol: funnel plot analysis

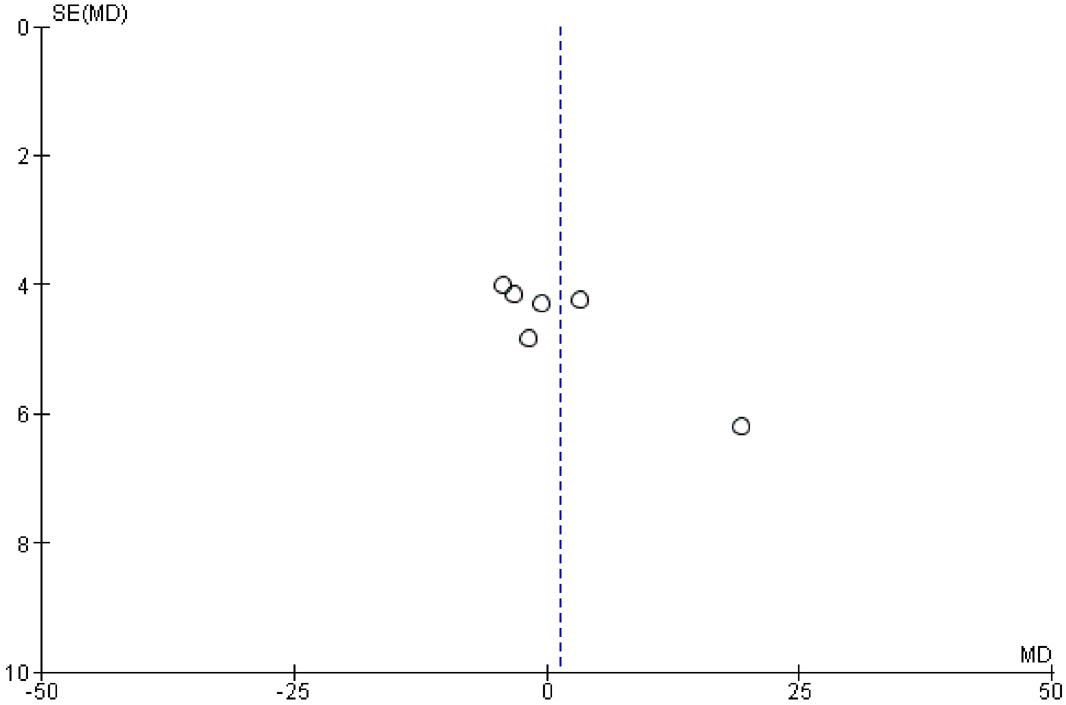
SE(MD)
0
2
4
6
8
10
-50
-25
0
25
50
MD
