# Supplementary material for: Dapagliflozin & pioglitazone combination therapy in T2DM with or without MASLD - a systematic review and meta-analysis: PRO-2 study
Source: Front Clin Diabetes Healthc. 2026 Jul 20;7:1733995. doi: 10.3389/fcdhc.2026.1733995 (PMC13429781; doi:10.3389/fcdhc.2026.1733995)
Supplement: Supplementary file 2 [file DataSheet2.docx]

**Appendix**

**Table A1.** Certainty of evidence (GRADE assessment)

| **Outcomes** | **Number of Studies** | **Risk of Bias** | **Inconsistency** | **Indirectness** | **Imprecision** | **Other Considerations** | **Participants (Intervention/Comparator)** | **Effect (Mean difference)** | **Certainty of Evidence (GRADE)** | **Importance** |
| --- | --- | --- | --- | --- | --- | --- | --- | --- | --- | --- |
| Body weight | 9 RCTs | Not serious | Serious^a^ | Not serious | Not serious | None | 1190/1361 | 2.88 less (95% CI: 5.38 to 0.38 less) | ⊕⊕⊕○  Moderate | Critical |
| HbA1c | 13 studies | Not serious | Serious^a^ | Serious^a^ | Not serious | None | 1411 (intervention) | 0.41 less (95% CI: 0.56 less to 0.26 less) | ⊕⊕⊕○  Moderate | Critical |
| Total cholesterol | 7 RCTs | Not serious | Serious^a^ | Not serious | Not serious | None | 689/665 | 3.62 more (95% CI: 3.07 less to 10.30 more) | ⊕⊕⊕○  Moderate | Important |

*Abbreviations:* CI (confidence interval). *Notes: Downgraded due to between-study heterogeneity and variation in study designs contributing to the pooled estimates.*


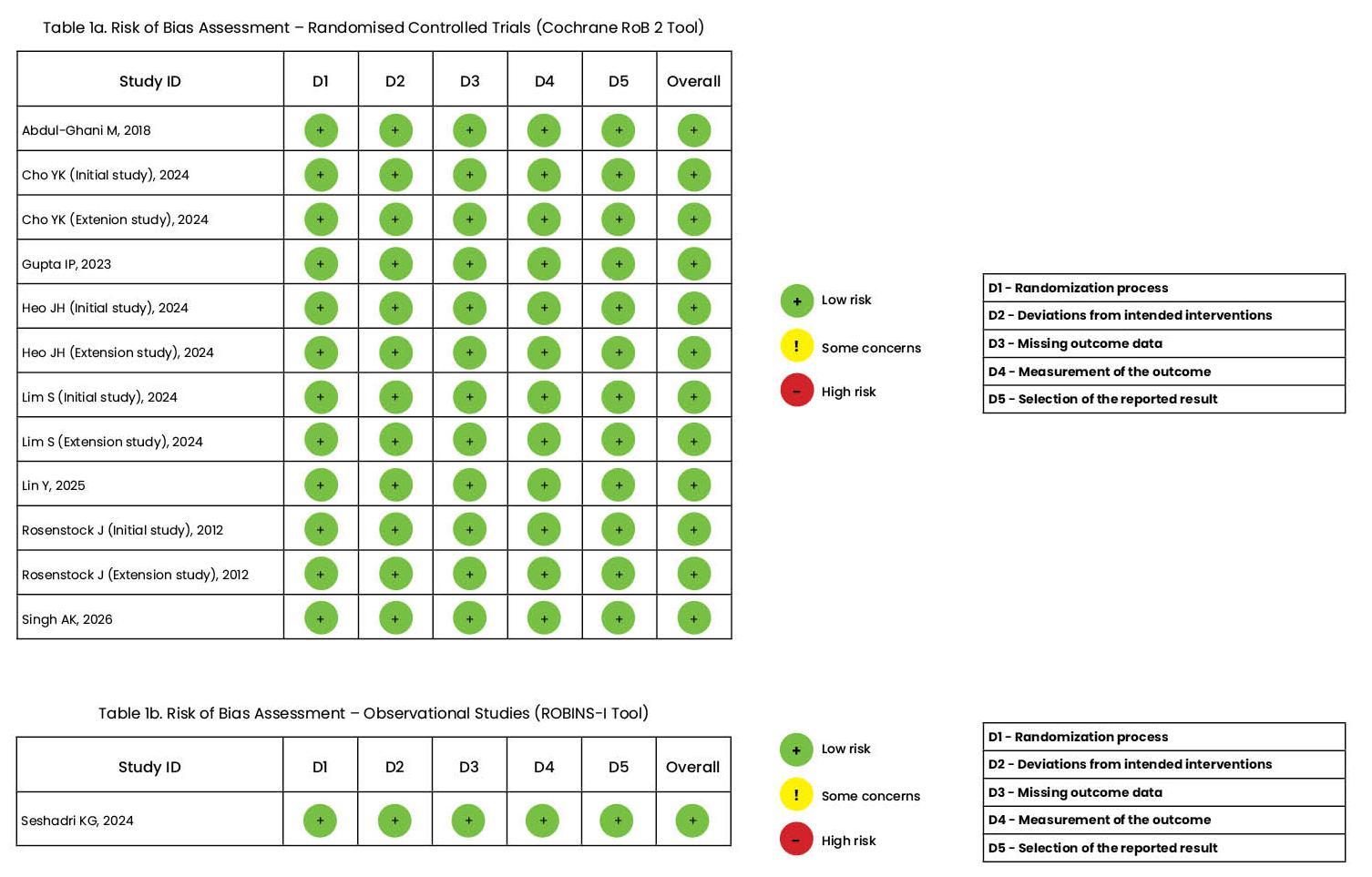


**Figure A1.** Risk of bias assessment: A) randomized controlled trials (RoB 2) and B) observational studies (ROBINS‑I).
